# Optimizing parameters of an open-source airway segmentation algorithm using different CT images

**DOI:** 10.1186/s12938-015-0060-2

**Published:** 2015-06-26

**Authors:** Pietro Nardelli, Kashif A Khan, Alberto Corvò, Niamh Moore, Mary J Murphy, Maria Twomey, Owen J O’Connor, Marcus P Kennedy, Raúl San José Estépar, Michael M Maher, Pádraig Cantillon-Murphy

**Affiliations:** School of Engineering , University College Cork, College Road, Cork, Ireland; Department of Respiratory Medicine, Cork University Hospital, Wilton, Cork, Ireland; Department of Radiology, Cork University Hospital, Wilton, Cork, Ireland; Department of Radiology, Brigham and Women’s Hospital, Harvard Medical School, Boston, MA USA

**Keywords:** Airway segmentation, Region growing, Computed tomography (CT), 3D Slicer, ITK, Image processing, Lung

## Abstract

**Background:**

Computed tomography (CT) helps physicians locate and diagnose pathological conditions. In some conditions, having an airway segmentation method which facilitates reconstruction of the airway from chest CT images can help hugely in the assessment of lung diseases. Many efforts have been made to develop airway segmentation algorithms, but methods are usually not optimized to be reliable across different CT scan parameters.

**Methods:**

In this paper, we present a simple and reliable semi-automatic algorithm which can segment tracheal and bronchial anatomy using the open-source 3D Slicer platform. The method is based on a region growing approach where trachea, right and left bronchi are cropped and segmented independently using three different thresholds. The algorithm and its parameters have been optimized to be efficient across different CT scan acquisition parameters. The performance of the proposed method has been evaluated on EXACT’09 cases and local clinical cases as well as on a breathing pig lung phantom using multiple scans and changing parameters. In particular, to investigate multiple scan parameters reconstruction kernel, radiation dose and slice thickness have been considered. Volume, branch count, branch length and leakage presence have been evaluated. A new method for leakage evaluation has been developed and correlation between segmentation metrics and CT acquisition parameters has been considered.

**Results:**

All the considered cases have been segmented successfully with good results in terms of leakage presence. Results on clinical data are comparable to other teams’ methods, as obtained by evaluation against the EXACT09 challenge, whereas results obtained from the phantom prove the reliability of the method across multiple CT platforms and acquisition parameters. As expected, slice thickness is the parameter affecting the results the most, whereas reconstruction kernel and radiation dose seem not to particularly affect airway segmentation.

**Conclusion:**

The system represents the first open-source airway segmentation platform. The quantitative evaluation approach presented represents the first repeatable system evaluation tool for like-for-like comparison between different airway segmentation platforms. Results suggest that the algorithm can be considered stable across multiple CT platforms and acquisition parameters and can be considered as a starting point for the development of a complete airway segmentation algorithm.

## Background

Computed tomography (CT) is a common imaging modality frequently required for diagnosis and assessment of lung disease [1, 2]. A CT dataset typically consists of a large number of images, requiring tedious inspection of individual slices for signs of disease. Moreover, first results from the lung cancer screening trial data show that around one third of smoking people that undergo a CT scan have lung nodules that may require guided bronchoscopy and biopsy [3]. For these reasons, automatic segmentation of the tracheal and bronchial anatomy followed by a 3D reconstruction may significantly improve the physician’s ability to assess pathological conditions. In particular, airway segmentation may help to form a pathway to a focal peripheral lesion as well as to visualise a focal airway problem such as a structure, or to create an airway splint [4]. Several techniques of airway segmentation starting from CT images have been proposed, but the problem of segmenting the narrow peripheral airways still represents a major technical challenge. These narrow outer airways are particularly susceptible to image-reconstruction artifacts, patient movements and partial volume effect which may introduce degradation.

Many airway segmentation techniques rely on a region-growing approach, a fast method that requires no prior knowledge of the structure of the airway, and uses a seed voxel and an intensity threshold to separate air from tissue voxels [5–9]. The main problem with this technique is leakage, caused by voxels misclassified as air voxels. Leakage causes the segmentation to extend outside the airway and leak into the lung parenchyma [7, 8].

To address this problem, several solutions have been proposed. One of the first methods, proposed by Mori et al. [7], used a 3D painting algorithm to directly extract the inside of the airway tree automatically increasing the intensity threshold until leakage occurs. Schlathölter et al. [8] implemented a fast marching algorithm in which wave-front propagation is used in conjunction with an anatomical model of the airway tree to implement a region growing algorithm, which ends when leakage occurs in all the directions of propagation. A different approach, based on stopping the segmentation when leakage occurs, was proposed by Kitasaka et al. [5]. In that approach, a given pre-segmented volume is divided into sub-parts, called Volumes of Interest (VOI), each containing a branch. These VOIs are then refined using Mori’s method [7]. Tschirren et al. [9], prompted by [5], proposed an algorithm which also uses VOIs of a previously segmented airway, but using the topology of thinned structures to avoid leakage. Lai et al. [6] extended the concept of VOI of [5] to produce Volume of Rough Segmented (VOR) parts. These are then divided into three different types based on their position in the airway, to be finely segmented using a specific method for each type. Lo et al. [10, 11] proposed an algorithm where an airway appearance model is used in combination with a vessel tree segmentation to develop a classifier able to automatically discern between airways and surrounding tissue voxels using local descriptors. Kiraly et al. [12] proposed an algorithm using a 3D region growing method based on Mori’s algorithm to segment larger airways, combined with specific morphologic operators to improve the segmentation. Salito et al. [13] applied an automated 3D region growing on healthy subjects and patients with severe emphysema, to evaluate the effect of emphysema on airway segmentation. In Gao et al. [14] a region growing approach is combined with a morphological gradient information to help the region growing further segment peripheral branches from surrounding tissue with similar intensity. Graham et al. [15–18] proposed a method in which a first adaptive region growing method similar to [7] is applied. Afterwards, branch segments are identified considering tube-like structures. Finally, neighbouring branch segments are connected by smooth interpolated surfaces. Irving et al. [19] extended the morphology based method proposed by Pisupati et al. [20], by including a three dimensional morphological filtering and leak removal using 3D dilation. Recently, a further extension of this approach has been presented [21], which proposes integration of the airway tree topology and branch shape to help identify and segment missing branches. This method is potentially applicable to most airway segmentation methods as a second step. Rudyanto et al. [22] presented an airway posterior probability model that exploits a novel multi-scale wallness measure to develop a probabilistic map that may help to optimize the cost function for region growing or fast marching-based algorithms. Rizi et al. [23] proposed a fuzzy connectivity region growing that exploits the cylindrical properties of the airway branches. This method seems to prevent leakage apperance. Zhu et al.[24] propose a method that employs an initial 3D region growing followed by a 3D wave propagation and a morphological filter to optimize segmentation. These steps are iterated several times until an accurate segmentation is found. Finally, in Xu et al. [25] a hybrid multi-scale approach that combines intensity-based region growing with morphology based method and a multi-scale vesselness to try to segment peripheral branches while avoiding leakage was presented.

Algorithms are commonly tested only on specific image types and their reliability is usually not proven across images acquired using different characteristics, such as slice thickness, reconstruction kernel and radiation dose. Furthermore, algorithms are normally not freely available. Therefore, the development of an open-source software platform for airway segmentation may help in comparing other methods with the one proposed in the present paper. An open-source platform also facilitates continuos improvement of the algorithm as new outcomes are obtained. Moreover, an open-source algorithm may be modified according to specific needs and purposes.

In this work, we describe a semi-automated algorithm for airway segmentation in CT images based on lung-side-specific region growing approach using the intensity range of pixels. The algorithm is implemented as an extension of the open-source software platform 3D Slicer [26] so that it can be easily downloaded and compared with other teams’ methods and can also be modified and improved according to different needs. The algorithm has been evaluated on human CT images, exploiting local images and cases from the publicly available EXACT’09 dataset [27]. To evaluate the reliability of the algorithm, images from the same subject scanned with different parameters have been investigated. As a further test, the method has been applied on a breathing pig lung model that was developed to evaluate the effect of different scanning parameters and to demonstrate the stability of the algorithm across different CT protocols and types of images. “Methods” details the proposed airway segmentation method. “CT data” focuses on the description of experimental data used for testing the algorithm. “Results and discussion” shows the results obtained and demonstrates the reliability of the algorithm. Finally, in “Conclusion” perspectives and conclusions are drawn.

## Methods

For the development of the algorithm, the freely available and easily extendible software platform, 3D Slicer, has been used [28–32]. The algorithm has been written mostly in C++ and Python, exploiting the functionality of Insight Segmentation and Registration Toolkit (ITK) [29, 30] and Visualization Toolkit (VTK) [32] classes. The proposed method is available as an extension of Slicer and can be downloaded and tested on personal datasets [33]. A video tutorial to show functionality of the module to end-users is also available online [34]. Since it has been written as an open-source module, the method can also be modified based on individual purposes and needs. To the best of our knowledge this is the first open-source algorithm entirely dedicated to airway segmentation. Other open-source tools, such as AirwayInspector [35] and PulmonaryToolkit [36] are available online, although oriented to quantitative analysis of the lung rather than to airway segmentation. The proposed method is based on a modified 3-D region growing algorithm, using an intensity threshold as an inclusion criteria. Only voxels having an intensity value below the specified threshold are considered part of the segmented region. However, noise may cause some airway-wall and voxels to be blurred and hence become no longer recognizable from the lung parenchyma, leading to leakage. For this reason, the threshold can be iteratively increased until leakage appears. Others parameters such as number of voxels considered to avoid leakage have been optimised based on robustness of results across all datasets. The approach presented here may stop segmentation too early, causing peripheral airways to be excluded from the segmented region. Therefore, we propose to subdivide the lung volume into three different parts; trachea, right and left lungs. This way, the three parts can be segmented separately, each using a different threshold which is optimal for avoiding local leakage and segmenting peripheral airways. An example of the optimal thresholds as identified by the software on some local clinical cases and on EXACT’09 [27] cases is reported in Table 1. Cropping of the volume and individual segmentation of the three parts are described in the following sections.

**Table 1:** Optimal thresholds (in HU) identified by the software for airway segmentation of human cases

	Trachea	Right lung	Left lung
CUH 1	−800	−800	−824
CUH 2	−930	−1,000	−1,024
CUH 3	−800	−1,096	−1,000
CUH 4	−800	−995	−976
Case 21	−800	−840	−820
Case 22^a^	−800	−1,008	−1000
Case 23	−820	−1,003	−996
Case 24	−820	−980	−974
Case 25^a^	−800	−955	−972
Case 26	−860	−925	−846
Case 27^a^	−800	−800	−800
Case 28	−800	−866	−835
Case 29^a^	−800	−912	−930
Case 30	−800	−825	−820
Case 31	−800	−958	−1,000
Case 32	−800	−997	−1,000
Case 33	−800	−953	−940
Case 34	−800	−1,000	−980
Case 35	−800	−918	−907
Case 36	−800	−851	−825
Case 37	−800	−889	−894
Case 38^a^	−800	−800	−800
Case 39	−800	−950	−959
Case 40	−800	−1,009	−1,005

Thresholds are chosen independently for trachea, right and left lungs.

^a^Scan was acquired from the same subject as the previous ones.

### Volume cropping and trachea segmentation

The segmentation method proposed here belongs to the group of method referred to as semi-automated, as it requires the user to manually place a seed within the trachea, which is easily recognizable in an axial CT image. Different approaches, such as De Nunzio et al. [37], automatically find the trachea in the first slices of the CT scan. However, the system here proposed is meant to be as general as possible, considering non-human images as well. As an example, for airways which have a branch from the trachea above the carina, as in the case of a pig lung, the seed point has to be placed between this branch and the carina. Therefore, we consider a manual placement of the seed point as a good trade-off between versatility and automation.

**Figure 1:**
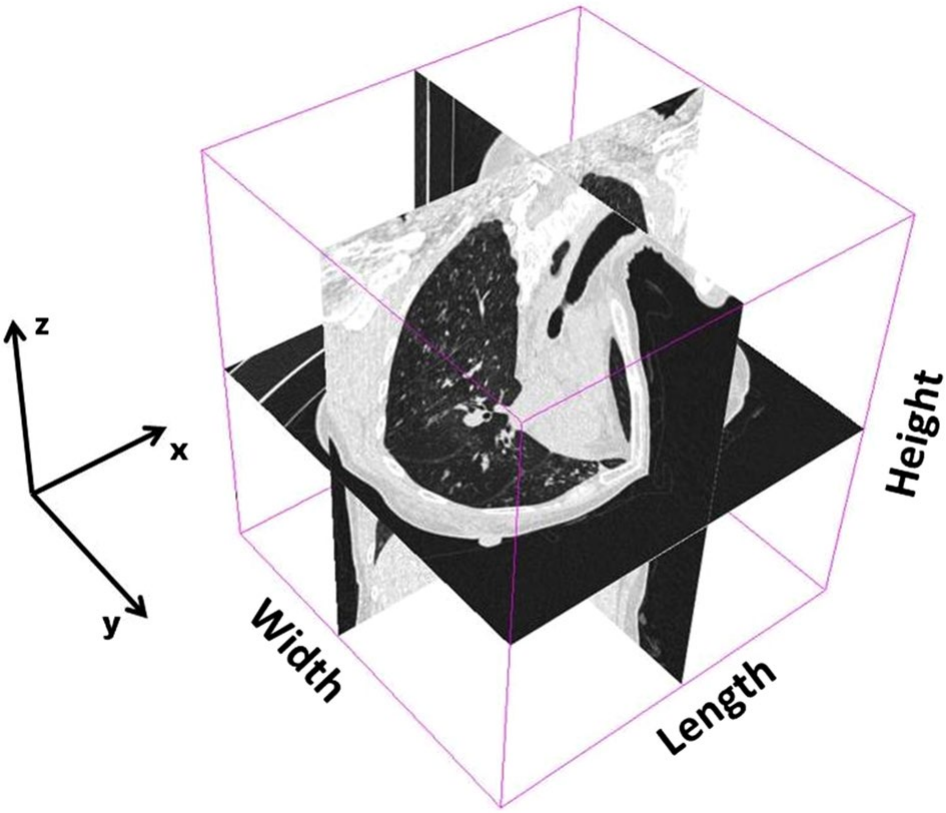
Representation of length, width and height in a 3D volume as utilised in this approach.

**Figure 2:**
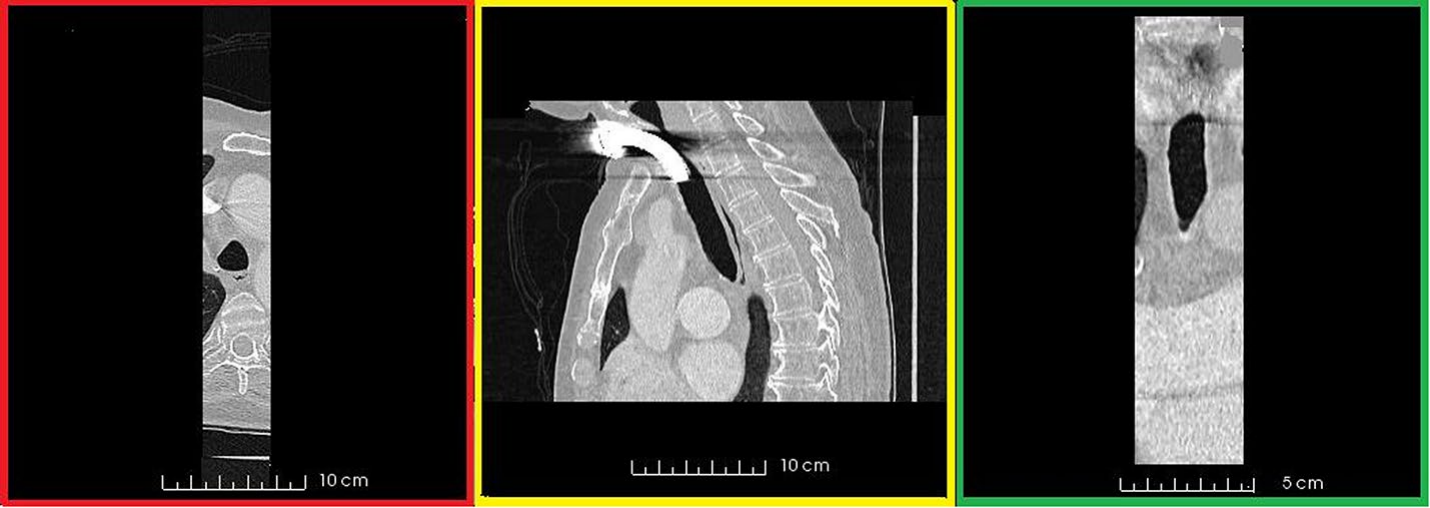
Representation of an axial (*red*), a sagittal (*yellow*), and a coronal (*green*) slice of the cropped volume. The whole volume is cropped around the trachea, considering almost the entire length volume. Cropping is refined after the first segmentation exploiting information on the carina position.

**Figure 3:**
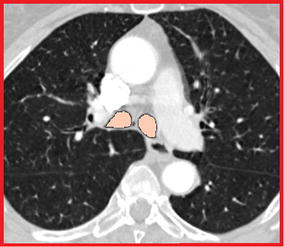
Axial slice containing the carina. The carina is found on the axial slice where the overlay trachea label splits into two different parts. This example was extracted from case 28 of the EXACT’09 project.

The algorithm’s first step involves cropping the whole volume in order to extract the trachea. To this end, an average trachea length, the whole volume width, and a height given by the whole volume minus a small portion of volume itself are considered (for definition of depth, width and length in a 3D volume see Figure 1). An example of the first cropped volume as extracted by the algorithm is given in Figure 2 where an axial, a sagittal, and a coronal slice view are shown. Using this cropped volume the initial segmentation of the trachea starting from the placed fiducial point is accomplished. Details of how trachea segmentation is performed are reported in the first sub-section. Once this first segmentation is completed, the second step involves using the obtained trachea label to improve the cropping of the trachea volume. To achieve this, the carina position is computed automatically, by scanning from the fiducial position and moving slice by slice towards the carina along the axial slices. The algorithm recognises the carina as the point in which the segmented label splits into two different parts, representing the two main bronchi. As an example, Figure 3 shows the carina position as found on a CT image after the first trachea segmentation. The algorithm’s third step uses the carina position to compute the maximum height of the trachea, and the volume is cropped accordingly. Simultaneously with this third step, the cropping size is also updated in length, to take into account possible bends in the trachea. This is achieved by moving slice by slice along the z axis and identifying points in which the label touches the side borders of the previously cropped volume, in which cases the cropping is extended in length. The fourth step involves a second and final trachea segmentation of the new cropped volume. Once the trachea label is finalised, the carina position within the trachea label is used to automatically define the seed points for the segmentation of the right and left lungs. Details of right and left lungs segmentation are reported in the second sub-section.

#### Trachea segmentation

The seed point placed by the user is exploited as starting point for the segmentation of the trachea. Starting from a value of −900 HU, the threshold is iteratively and automatically increased until it either reaches the maximum value of −800 HU or leakage occurs. At the initial threshold the volume is segmented and the width of the obtained trachea’s label is computed. To this end, a small region of the axial section of the label around the seed point’s position is extracted and the width calculated. Simultaneously, a small set of coronal images of the label is also extracted and its length computed. As a check on the subsequent automatic segmentation of the trachea, these computed values are compared with the entire cropped volume of the trachea. If the label has a coronal length of less than two thirds of the whole cropped volume and an axial width less than one quarter of the whole cropped volume, it is assumed that no leakage has occurred and the two label sizes are stored. Otherwise, the threshold is repeatedly decreased in increments of 20 HU until leakage is no longer evident. On the other hand, if −900 HU is not high enough as an initial threshold to obtain trachea segmentation, voxels around the seed point are first evaluated as new possible starting points, and if none of the 26 touching voxels gives a segmentation, the threshold is increased in increments of 50 HU and the previously described process repeated. At this point, the threshold is iteratively increased to check whether it is possible to obtain improved segmentation, that is segmentation of more peripheral branches without leakage appearance. Again, if no leakage has occurred in the previous step, 50 HU is added to the threshold. If leakage has previously occurred 10 HU is added. Hence, a new segmentation is computed and the old and new labels are subtracted. In this way, differences referring to how much trachea label has been added using the new threshold can be calculated. Width and length of these differences are computed and compared with the previously stored values. This process is repeated until either the size of the added labels is larger than the memorized sizes or the height of the label is less than one third of the height of the trachea volume. If the previous conditions on size of the added label are not satisfied, leakage is likely to occur. In this case, the threshold is repeatedly decreased in increments of 10 HU until a label with no leakage is obtained.

#### Right and left lungs segmentation

**Figure 4:**
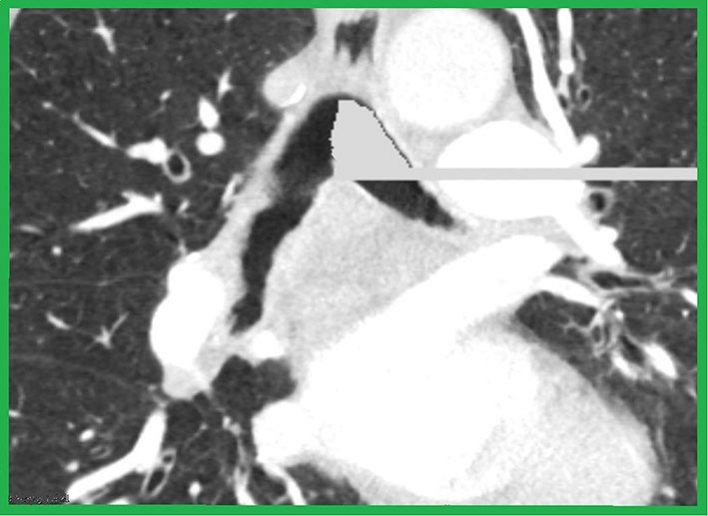
Example of “masking” as it appears on a sagittal slice. Half trachea label is used to turn values where the half trachea overlays to 0 HU (*grey voxels*). In the picture, the left part of the trachea is masked, to allow segmentation of the right lung. A closing process is also used to make sure that no spreading within the left lung is obtained.

**Figure 5:**
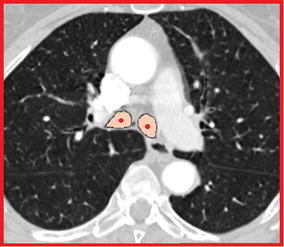
Fiducial points representation as automatically identified and placed on the carina. This example was extracted from the same EXACT’09 case as in Figure 3.

The right and left lungs have to be separated and segmented. In order to do so, the algorithm uses half of the trachea label obtained to ”mask” the trachea in the original CT volume. In particular, the half trachea distal from the lung to be segmented is considered. Therefore, in the original image the intensity value of all the “masked” voxels inside the trachea is set to 0 HU, a value much higher than the threshold that will be used for the airway segmentation. In this way, it is not necessary to crop the volume again with the segmentation still limited to only one lung at the time. Figure 4 illustrates how the left half of the trachea is “masked” to segment the right lung. The grey part represents voxels that have been given a 0 HU value. As shown in the picture, a method to “close” the opposite main bronchus has also been implemented, so that segmentation of one lung does not spread within the other one. Using half of the trachea label to mask the trachea at this stage facilitates segmentation of animal lungs, such as the pig lung, where a tracheal branch may be present above the carina. Obviously, this branch would not be segmented during the trachea segmentation, because of the volume cropping, and this would lead to an incomplete segmentation. With the method we propose, the segmentation is spread into part of the trachea itself allowing the segmentation of any possible branch above the carina position. Regarding the starting points to be used for segmentation, two seed points are automatically defined on the axial slice containing the carina in the central point of the two parts into which the label splits, as shown in Figure 5.

As for the trachea, segmentation of the right and left lungs uses a 3D region growing method with an iteratively increased threshold. However, in this case leakage occurrence is controlled by two approaches, based on Tschirren et al. [38]. Firstly, any sudden increase in the number of voxels between segmentations with two consecutive thresholds is considered leakage. Secondly, a maximum number of allowed voxels for the segmentation is also defined. In order to define when the increase between two successive segmentation steps is big enough to lead to leakage, a value of

$$\begin{aligned}g = \frac{{N_{{{\text{voxels}}}} }}{{N_{{{\text{voxels}}\_{\text{prev}}}} }}\end{aligned}$$

where N_voxels_ is the number of voxel of the actual segmentation, while N_voxels_prev_ represents the number of segmented pixel of the previous segmentation, is computed and compared to g_max_ = 1.6, as in [38]. A value of N_voxels_max_ = 500,000 for maximum number of voxels allowed in the segmentation is proposed in [38], while in other works such as [12] and [17], a maximum volume of V_e_ = 50,000 mm^3^ and V_e_ = 75,000 mm^3^, respectively, was defined. Since we do not consider the entire lung for segmentation, but only part of it, a novelty part idea that we propose is that N_voxels_max_ be related to the number of voxels of the trachea. In particular, we use a specific percentage of the trachea number of voxels, based on the size of the trachea. This percentage can be optimized according to the different characteristics of the dataset under inspection. Table 2 summarizes the different percentages depending on the CT characteristics, such as reconstruction kernel and slice thickness. These values have been empirically calculated from the datasets available to date. Also, a general percentage for not yet inspected datasets is provided. In this sense, since datasets acquired with all the possible combination of parameters were not available, percentages have been optimized for the types of datasets available for the time being. However, since our system is open-source, parameters will be continuously updated and optimized when new datasets are considered. As showed in the results section, the chosen percentage values are quite appropriate to achieve a good trade-off between leakage and depiction of segmented peripheral branches and provide a reliable method across different CT scan images acquired with different characteristics. Finally, once the right and left lungs’ labels are obtained, they are merged with the trachea’s segmentation to create a unique inseparable airway label. In some cases, the label might present some disconnected parts. Therefore, as a final step a hole filling and dilation steps are used to connect these potentially disconnected parts.

**Table 2:** Percentage of trachea voxels used for different reconstruction kernels and slice thicknesses

# of slices (S)	# of voxels (N)	Percentage
STD, B20f, B30f, B, C, FC10, FC12^a^		
$S \le 300$	N $>$ $5\times 10^4$	0.5
	$2\times 10^4$ $<$ $N \le 5\times 10^4$	0.75
	$N \le 2\times 10^4$	0.9
300 $<$ $S \le 400$	N $>$ $10^5$	0.5
	$8.5\times 10^4$ $<$ N $\le \ 10^5$	0.75
	$N \le 8.5\times 10^4$	0.9
S $>$ 400	N $>$ $17\times 10^4$	0.5
	$14\times 10^4$ $<$ $N \le 17\times 10^4$	0.75
	$N \le 14\times 10^4$	0.9
LUNG, B50f, FC50, FC52^b^		
$S \le 300$	N $>$ $8.5\times 10^4$	0.2
	$7.5\times 10^4$ $<$ $N \le 8.5\times 10^4$	0.3
	$3.5\times 10^4$ $<$ $N \le 7.5\times 10^4$	0.35
	$10^4$ $<$ $N \le 3.5\times 10^4$	0.5
	$N \le 10^4$	0.8
300 $<$ $S \le 400$	N $>$ $12\times 10^4$	0.2
	$10^5$ $<$ $N \le 12\times 10^4$	0.35
	$8.5\times 10^4$ $<$ $N \le 10^5$	0.5
	$N \le 8.5\times 10^4$	0.7
S $>$ 400	N $>$ $14\times 10^4$	0.2
	$11.5\times 10^4$ $<$ $N \le 14\times 10^4$	0.35
	$8\times 10^4$ $<$ $N \le 11.5\times 10^4$	0.5
	$N \le 8\times 10^4$	0.75
B60f, B70f, B70s, D^c^		
$S \le 300$	N $>$ $9\times 10^4$	0.35
	$6\times 10^4$ $<$ $N \le 9\times 10^4$	0.5
	$3\times 10^4$ $<$ $N \le 6\times 10^4$	0.6
	$N \le 3\times 10^4$	0.8
S $>$ 300	N $>$ $12\times 10^4$	0.25
	$8\times 10^4$ $<$ $N \le 12\times 10^4$	0.4
	$5\times 10^4$ $<$ $N \le 8\times 10^4$	0.6
	$N \le 5\times 10^4$	0.8
Any other Kernel^d^		
$S \le 300$	N $>$ $9\times 10^4$	0.35
	$4\times 10^4$ $<$ $N \le 9\times 10^4$	0.55
	$N \le 4\times 10^4$	0.8
S $>$ 300	N $>$ $13\times 10^4$	0.3
	$7\times 10^4$ $<$ $N \le 13\times 10^4$	0.55
	$N \le 7\times 10^4$	0.8

S is used to take into account the different slice thicknesses.

^a^Kernels: GE Medical System Standard, Siemens B20f and B30f, Philips B and C, Toshiba FC10 and FC12.

^b^Kernels: GE Medical System Lung, Siemens B50f, Toshiba FC50 and FC52.

^c^Kernels: Siemens B60f, B70f and B70s, and Philips D.

^d^Any other kernel.

#### Leakage evaluation method

**Figure 6:**
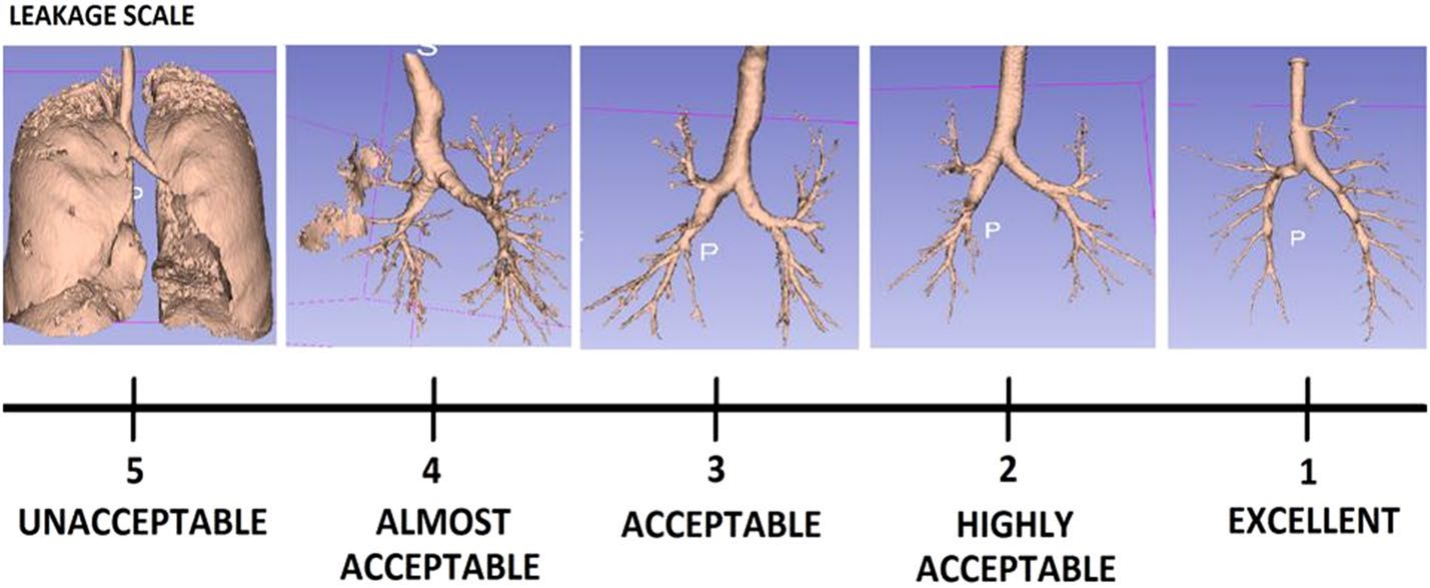
Leakage scoring scheme as presented to clinical experts. A score of *5* is given to an image with significant leakage presence, while *1* represents an image with no leakage.

Leakage presence is the most important parameter to be considered once airway is segmented. This often turns out to be a complicated task, as it may be difficult to distinguish a small leakage from a correctly segmented branch. For this reason, a new leakage evaluation system has been implemented for the presented work; four expert clinicians from the field of respiratory medicine or radiology were instructed on what leakage is. They were then asked to analyze the 3-D reconstructed model of the airway as well as the label placed on the chest CT image and to score the segmentation ranging from 1 to 5, where 5 was a segmentation presenting significant leakage and 1 was an image with no leakage. Figure 6 shows the scoring scheme presented to the clinicians in order to score the images. Average scores were then used to evaluate the segmentation.

**Figure 7:**
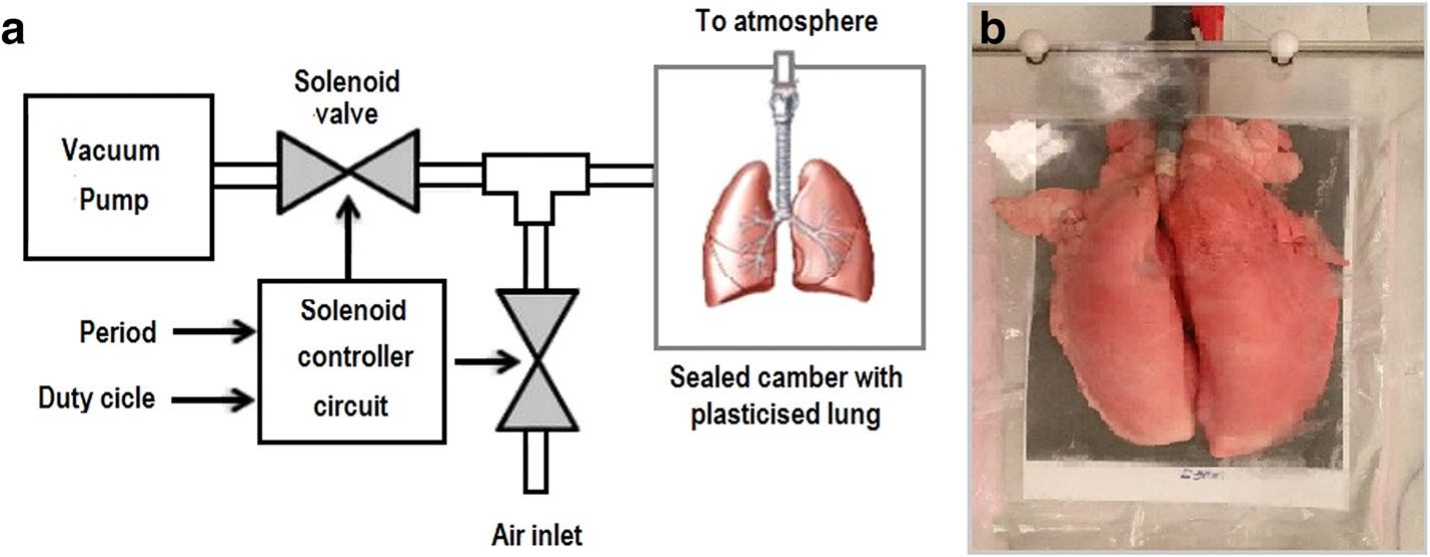
Breathing pig lung model. **a** Scheme representing the breathing pig lung model.** b** Plasticised pig lung when fully inflated.

## CT data

Many airway segmentation algorithms in the literature have been tested only on specific types of images, making evaluation of reliability across different platforms problematic. Parameters such as slice thickness, reconstruction kernel and radiation dose greatly affect the quality of the image, and thus the quality of segmentation may vary hugely. For these reasons, we have tested our algorithm on twenty four human cases and several breathing pig lung CT scans across scan parameters; slice thickness, convolution kernel, and radiation dose have been considered. For the human cases, we used the twenty test cases from the EXACT’09 dataset [27] and we compared our method with those of other teams who participated to the challenge. From the twenty test cases of the challenge we then extrapolated six cases belonging to the same subject, reconstructed using different parameters. We also tested the algorithm on four local clinical cases belonging to different subjects but reconstructed with the same convolution kernel. On the other hand, the pig scans were all acquired from the same model. Convolution kernel, slice thickness and radiation dose were varied for each scan. Images from the pig lung model were acquired during simulated inspiration, expiration and half inflation. The following two sub-sections detail the human data and the breathing pig lung model, respectively.

### Human cases

Four clinical chest CT scans were acquired using a GE Medical System scanner from four different subjects. The first two scans were acquired using a Discovery CT750 HD CT scanner, case 3 was scanned using a GE LightSpeed VCT scan, whereas the manufacturer model for case 4 was GE Discovery STE. The four patient scans were all provided by the Cork University Hospital (CUH) in compliance with an approved ethical protocol by the Cork Research Ethics Commitee. They all belong to patients with suspicious lung cancer and were selected from a regional multidisciplinary thoracic oncology meeting. Datasets were generated using a standard scanning protocol for lung cancer patients, i.e., asking the patient to hold a full-inspiration breath for less than 20 s to reduce motion artifacts. A voltage of 120 kVp was used for all cases, while the tube current varied from 60 to 200 mA as determined by automatic tube current modulation. Slice thickness was 1.25 mm for all the datasets. The final 3D images were all reconstructed using a lung convolution kernel. Table 3 reports acquisition parameters for the CUH cases.

**Table 3:** Acquisition parameters of the scans provided by CUH

	Manufacturer	Model name	Slice thickness (mm)	Kernel	Tube voltage (kVp)	Tube current (mA)	I\E
CUH 1	GE system	Discovery CT750 HD	1.25	LUNG	120	65	I
CUH 2	GE system	Discovery CT750 HD	1.25	LUNG	120	125	I
CUH 3	GE system	LightSpeed VTC	1.25	LUNG	120	60	I
CUH 4	GE system	Discovery STE	1.25	LUNG	120	170	I

*I/E* full-inspiration (I) or full-expiration (E) breath-hold.

In order to evaluate the algorithm on a larger database, we also took part in the the EXACT’09 challenge [27]. The datasets are publicly available and Table 4 presents acquisition parameters of the twenty test cases. Numbers of the cases represent the indices from the EXACT ’09 project.

**Table 4:** Acquisition parameters of the twenty cases from the EXACT’09 dataset

	Manufacturer	Model name	Slice thickness (mm)	Kernel	Tube voltage (kVp)	Tube current (mA)	I\E
Case 21	Siemens	Sensation 64	0.6	B50f	120	200.0	E
Case 22^a^	Siemens	Sensation 64	0.6	B50f	120	200.0	I
Case 23	Siemens	Sensation 64	0.75	B50f	120	200.0	I
Case 24	Toshiba	Aquilion	1.0	FC12	120	10.0	I
Case 25^a^	Toshiba	Aquilion	1.0	FC10	120	150.0	I
Case 26	Toshiba	Aquilion	1.0	FC12	120	10.0	I
Case 27^a^	Toshiba	Aquilion	1.0	FC10	120	150.0	I
Case 28	Siemens	Volume Zoom	1.25	B30f	120	348.0	I
Case 29^a^	Siemens	Volume Zoom	1.25	B50f	120	348.0	I
Case 30	Philips	Mx8000 IDT 16	1.0	D	140	120.0	I
Case 31	Philips	Mx8000 IDT 16	1.0	D	140	120.0	I
Case 32	Philips	Mx8000 IDT 16	1.0	D	140	120.0	I
Case 33	Siemens	Sensation 16	1.0	B60f	120	103.6	I
Case 34	Siemens	Sensation 16	1.0	B60f	120	321.0	I
Case 35	GE	LightSpeed 16	0.625	Standard	120	411.5	I
Case 36	Philips	Brilliance 16P	1.0	C	120	206.0	I
Case 37	Philips	Brilliance 16P	1.0	B	140	64.0	I
Case 38^a^	Philips	Brilliance 16P	1.0	C	120	51.0	E
Case 39	Siemens	Sensation 16	1.0	B70f	100	336.7	I
Case 40	Siemens	Sensation 16	1.0	B70s	120	90.6	I

* I/E* full-inspiration (I) or full-expiration (E) breath-hold.

^a^Scan was acquired from the same subject as the previous one.

Since our goal was also to compare clinical images reconstructed with different kernels but acquired with other CT platforms, we further extrapolated and considered six scans from the twenty cases of the EXACT’09 project [27]. In particular, we chose three scans belonging to the same subject and acquired with the same scanner, but using different parameters. We examined case 24 and 25 scanned with Toshiba Aquilion, case 28 and 29 acquired using a Siemens Volume Zoom scanner and case 37 and 38 that were obtained using a Philips Brilliance 16P scan.

### Breathing pig lung model

To further investigate reliability of the algorithm, a breathing pig lung model was developed. To this end, a BioQuest Inflatable Lung kit (Nasco, Fort Atkinson, WI, USA) was used as a phantom for CT image acquisition (see Figure 7a). This kit consists of plasticised pig lungs (Figure 7b) which can be inflated to various levels as required.

**Figure 8:**
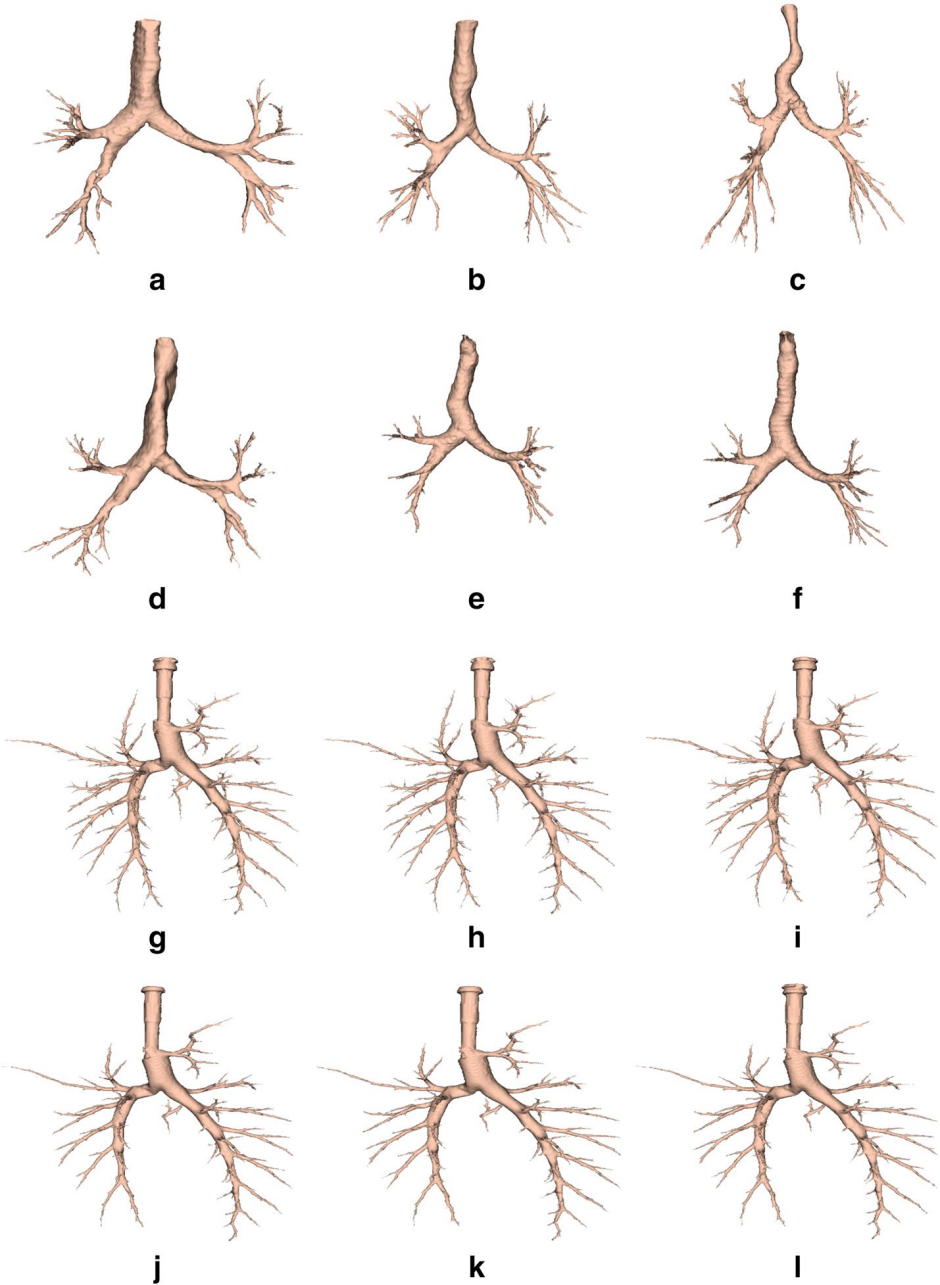
3D representations of results. Results have been obtained from **a**–**d** CUH (acquired with 1.25 mm slice thickness), from **e**,** f** EXACT’09 cases 24 and 25 (1.0 mm slice thickness), and from **g**–**l** six pig lung breathing model cases acquired using 0.625 mm slice thickness, during inspiration.** g**–**i** Reconstructed using a lung convolution kernel, varying dose from high to low, respectively.** j**–**l** Reconstructed with a standard convolution kernel, varying dose from high to low, respectively.

The lungs are placed in a vacuum chamber, with the trachea connected to atmospheric pressure. When the chamber is evacuated, the pressure differential between the outside and the inside of the lungs causes them to inflate. Venting the chamber to the atmosphere equalises the pressure which causes the lungs to collapse to an uninflated equilibrium form. The lungs were made to inflate and deflate in a programmable way to simulate standard breathing patterns. An Arduino Uno microcontroller was used to enable a set of solenoid valves (AD612 by CS Fluid Power) to control the lung inflation level. One valve connects to the vacuum pump, while another is used for venting the chamber. To set the breathing cycle, two dials are connected to the microcontroller. One dial controls the overall period of the cycle while a second one sets the inflation time as a percentage of the period. This simple and low cost solution proved very effective in simulating the human breathing pattern. Using this model, 48 CT scans of the lungs were generated during inspiration, expiration and half inflation phases. For both inspiration and expiration eight different protocols were used to evaluate the effect on image quality. The scanner used to get computerized tomographic images was GE Medical System Discovery CT750 HD. The reconstruction kernel was varied between lung and standard kernel and images were acquired at four different types of slice thickness ranging from 5 to 0.625 mm. Three different levels of tube current and radiation exposure have been used; a high radiation dose ranging from 60 to 160 mA, a medium dose of 40 mA and a low dose of 10 mA. Finally, an image at half inflation was acquired with a 0.625 mm slice thickness, high radiation dose and a standard reconstruction kernel to investigate the effect of lung inflation.

### Test procedure

To evaluate the performance of the algorithm, airway labels have been generated from all the datasets described. To this end, one seed point has been manually placed in the trachea of each case exploiting the 3D Slicer fiducial panel. For the pig lung model images, the seed point has been carefully placed between the additional branch coming out of the trachea and the carina. Then, the method has been invoked, and the labels generated. The segmentation process takes in average 2–3 min to complete on a 64-bit, i7-3770, 8 GB computer. From the generated labels, 3D models of the airways have been created leveraging the 3D Slicer model maker module, with parameters optimized for good visual results. No pre-filtering was used before the segmentation. Only in one case (for the pig breathing model that was scanned during inflation, with a 1.25 mm slice thickness, high radiation dose, and reconstructed with a lung kernel) a blur gaussian filtering was necessary to avoid a leakage “explosion”.

## Results and discussion

The first sub-section shows results for the proposed airway segmentation algorithm on human cases, while the second one reports results obtained using the breathing pig lung model. For evaluation of the EXACT’09 cases, the metrics as computed by the EXACT’09 authors have been considered. For the remaining clinical cases and the pig lung model cases branch count, branch length, airway volume and leakage score were calculated and evaluated. For the branch count, correctly segmented branches were counted by visual inspection. The centerline of the airway label was then extracted exploiting the classes provided by the Vascular Modeling Toolkit (vmtk) [39] and this was used to compute the length of the branches. The sum of all branch lengths of a case was considered to compute the final branch length of that case. Airway volume was computed considering the number of label voxels combined with voxel geometry. Finally, the most important parameter, the leakage score, was used to determine the quality of the segmentation for each image. To this end, the evaluation system described in the methods section has been used. Figure 8a–f show the 3D reconstruction obtained for six clinical cases. Figure 8g–l represent results obtained for six pig cases acquired during inspiration, with a 0.625 mm slice thickness, and varying the radiation dose. Cases shown in Figure 8g–i were reconstructed using a lung convolution kernel, whereas cases presented in Figure 8j–l were reconstructed with a standard convolution kernel.

**Figure 9:**
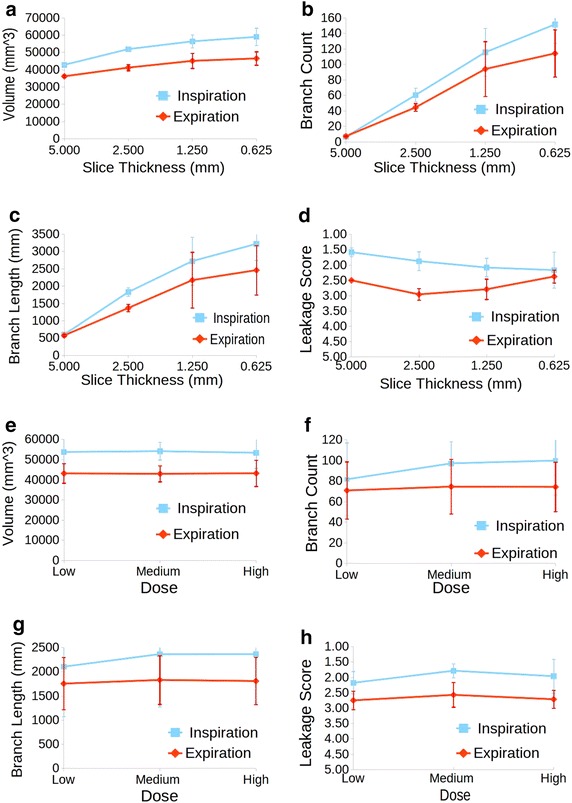
Results for the breathing pig lung model. Results are reported for inspiration (*blue*) and expiration (*red*), when varying **a**–**d** slice thickness and **e**–**h** radiation dose.

**Figure 10:**
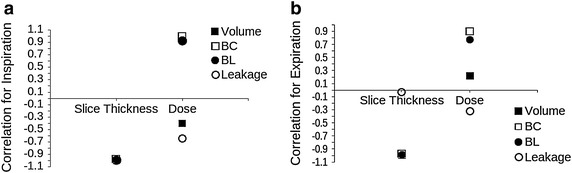
Correlation between image quality and metrics for the breathing pig lung model.** a** Inspiration phase.** b** Expiration phase.

### Results for human cases

In Table 1 the optimal thresholds identified by the software for the human cases are reported. As shown in the table, in most of the cases a different threshold is chosen for the segmentation of the two airways. Therefore, having a different threhsold for the trachea, the right and left lungs helps to have a better segmentation in one lung, that will not be affected by the presence of leakage in the other.

**Table 5:** Results obtained from the twenty test cases of the EXACT’09 project

	Branch count	Branch detected (%)	Tree length (cm)	Tree length detected (%)	Leakage count	Leakage volume (mm^3^)	False positive rate (%)
Case 21	98	49.2	51.1	46.2	1	0.2	<0.01
Case 22	141	36.4	98.6	29.8	13	77.7	0.60
Case 23	126	44.4	90.5	34.8	12	119.8	1.02
Case 24	72	38.7	57.0	35.1	23	245.9	1.82
Case 25	108	46.2	82.7	32.8	5	29.2	0.20
Case 26	32	40.0	21.6	32.8	2	226.3	7.53
Case 27	41	40.6	30.1	37.1	0	0.0	0.00
Case 28	69	56.1	47.7	43.6	0	0.0	0.00
Case 29	93	50.5	62.2	45.0	1	9.0	0.12
Case 30	79	40.5	57.0	37.3	0	0.0	0.00
Case 31	99	46.3	70.3	40.0	2	73.9	0.74
Case 32	89	38.2	73.2	33.6	2	29.9	0.26
Case 33	85	50.6	62.1	42.2	0	0.0	0.00
Case 34	264	57.6	195.7	54.7	16	89.5	0.41
Case 35	146	42.4	108.9	35.2	1	23.7	0.19
Case 36	121	33.2	122.8	29.8	2	2.6	0.03
Case 37	64	34.6	54.4	30.6	1	2.7	0.03
Case 38	37	37.8	27.3	41.1	3	4.0	0.08
Case 39	113	21.7	97.6	23.8	5	92.0	1.01
Case 40	102	26.2	91.2	23.6	5	21.5	0.17
Mean	99.0	41.6	75.1	36.5	4.7	52.4	0.71
SD	50.3	9.0	39.4	7.6	6.3	73.3	1.67
Min	32	21.7	21.6	23.6	0	0.0	0.00
1st quartile	69	36.4	51.1	30.6	1	0.2	<0.01
Median	96	40.6	66.2	35.1	2	22.6	0.18
3rd quartile	126	50.5	98.6	43.6	12	92.0	1.01
Max	264	57.6	195.7	54.7	23	245.9	7.53

Table 5 shows the results obtained from the EXACT’09 challenge. Considering the “mean” row of the table, as compared to the other teams’ mean results reported in Table 6, it is evident that the method is comparable to other methods in terms of branch count and tree length, and it is good in terms of leakage count and leakage volume as well as false positive rate. In choosing a threshold for the two lungs there is a good trade-off between airway segmentation and leakage presence.

**Table 6:** Mean results of the other teams taking part in the EXACT’09 challenge

	Branch count	Branch detected (%)	Tree length (cm)	Tree length detected (%)	Leakage count	Leakage volume (mm^3^)	False positive rate
Team 1	91.1	43.5	64.6	36.4	2.5	152.3	1.27
Team 2	157.8	62.8	122.4	55.9	12.0	563.5	1.96
Team 3	74.2	32.1	51.9	26.9	4.2	430.4	3.63
Team 4	186.8	76.5	158.7	73.3	35.5	5138.2	15.56
Team 5	150.4	59.8	118.4	54	1.9	18.2	0.11
Team 6	77.5	36.7	54.4	31.3	2.3	116.3	0.92
Team 7	146.8	57.9	125.2	55.2	6.5	576.6	2.44
Team 8	71.5	30.9	52	26.9	0.9	126.8	1.75
Team 9	139	56	100.6	47.1	13.5	368.9	1.58
Team 10	79.3	32.4	57.8	28.1	0.4	14.3	0.11
Team 11	93.5	41.7	65.7	34.5	1.9	39.2	0.41
Team 12	130.1	53.8	95.8	46.6	5.6	559	2.47
Team 13	152.1	63	122.4	58.4	5	372.4	1.44
Team 14	161.4	67.2	115.4	57	44.1	1873.4	7.27
Team 15	148.7	63.1	119.2	58.9	10.4	158.8	1.19

Table 7a reports the results obtained for the CUH datasets. In this case, the goal was to evaluate the reliability of the developed software across different human cases. For this reason, the most important metric to be considered is the leakage scoring, as for the others parameters no gold standard is available. As shown in the table, the average score for all the cases is close to 3, meaning that the clinicians considered the segmentation and the 3D reconstruction acceptable in terms of leakage presence.

**Table 7:** Results obtained for human cases

	Volume (mm^3^)	Branch count	Branch length (mm)	Leakage score
(a) Cases provided by CUH				
CUH 1	41,270.59	84	1,369.69	3 ± 1.15
CUH 2	34,772.03	106	1,505.25	2.50 ± 0.58
CUH 3	41,369.35	96	1,610.39	3.25 ± 0.50
CUH 4	58,288.49	88	1,425.97	3.25 ± 0.50

	Branch count	Branch detected (%)	Tree length (cm)	Tree length detected (%)	Leakage count	Leakage volume (mm^3^)	False positive rate	Leakage score
(b) Cases selected from the EXACT’09 cases								
Case 24	72	38.7	57.0	35.1	23	245.9	1.82	2.75 ± 0.50
Case 25^a^	108	46.2	82.7	32.8	5	29.2	0.20	2.50 ± 0.58
Case 28	69	56.1	47.7	43.6	0	0.0	0.0	3.25 ± 0.50
Case 29^a^	93	50.5	62.2	45.0	1	9.0	0.12	3.50 ± 0.58
Case 37	64	34.6	54.4	30.6	1	2.7	0.03	2.50 ± 0.58
Case 38^a^	37	37.8	27.3	41.1	3	4.0	0.08	3.00 ± 0.00

^a^Scan was acquired from the same subject as the previous ones.

As a further test on clinical cases, the effect of changing parameters during scan acquisition on airway segmentation was assessed on six EXACT’09 cases. Table 7b shows the results for the selected cases. Results are the same as reported in Table 5 with an added column representing the leakage score as evaluated by the four clinicians. As for leakage appearance, the segmentation does not seem to be particularly affected by variation of the parameters. However, comparing case 28 and 29, that where acquired changing only the convolution kernel, the resultant airway volume, branch count, and branch length were slightly different between the two cases. In particular, the use of a sharper B50f kernel gives a better segmentation than using a smoother B30f kernel. In terms of leakage, both cases were considered quite acceptable, although case 28 scored slightly better. This result was quite expected, as the use of different kernels affects the quality of the image. In particular, a sharper kernel would preserve higher spatial frequencies at the expense of greater image noise, whereas a smoother kernel would decrease noise and spatial resolution, reducing at the same time the higher frequency contribution. Therefore, a sharper kernel allows a more peripheral aiway segmentation, at the cost of a bit more leakage. In fact, the size of peripheral branches decreases going deeper in the lung, leading to a blurring effect that makes peripheral branches less recognizable from the lung parenchyma.

EXACT’09 cases 24 and 25 were also reconstructed using two different kernels. In particular, a smoother FC10 kernel was used for case 25. Therefore, as for the previous comparison, a better segmentation may be expected for case 24. However, as shown in Table 4, case 25 was acquired using a higher radiation dose. This affects the quality of the image more than kernel variation, leading to a far better segmentation in terms of airway volume, branch count, and branch length. Furthermore, the leakage score was also slightly lower for case 25. In this case, this may be due to improved quality of the image which at the same time improved the quality of peripheral branches. Note that for cases 26 and 27 (acquired and reconstructed with the same parameters as case 24 and 25, respectively) a similar situation occurs, confirming the results for the previous 2 cases. Since their reconstruction parameters were the same as of case 24 and 25 these cases were not reported in the table. Analysis of segmentation for cases 37 and 38 is slightly different. Here, case 37 was scanned using a smoother kernel, but with a higher radiation dose and more importantly a full-inspiration breath-hold, while case 38 was acquired during a full-expiration breath-hold. In this latter case, a full-inspiration breath-hold guarantees that more airway branches will be visible on the CT image, since branches will be more expanded and the air inside them will help to increase the contrast with the lung tissue. For this reason, far better results are obtained for case 37 compared to case 38. This may also explain the difference in the leakage score. It should be noted that the CT datasets from EXACT’09 were only used for comparative analysis of segmentation parameters, rather than comparison with any gold-standard. In general, results obtained on human datasets are promising and show that the proposed algorithm with the optimized parameters is quite stable across variation of scanning parameters. However, to avoid leakage segmentation in some cases the proposed algorithm may stop too early, avoiding possible segmentation of peripheral branches. In this sense, a new method for segmentation of peripheral branches to be integrated with the proposed algorithm may be of great help and will be considered for future improvement.

**Table 8:** Results obtained on the pig lung model for kernel variation for inspiration and expiration

Kernel	Volume (mm^3^)		Branch count		Branch length (mm)		Leakage score	
	Inspiration	Expiration	Inspiration	Expiration	Inspiration	Expiration	Inspiration	Expiration
Standard	53,025.54	41,213.01	91.89	52.22	2,240.55	1,512.20	1.89 ± 0.28	2.67 ± 0.35
Lung	54,374.74	44,516.82	94.73	89.00	2,320.74	2,008.65	2.04 ± 0.50	2.69 ± 0.32

### Results for breathing pig lung model

The breathing pig lung model has been used to evaluate the effect of slice thickness, reconstruction kernel and radiation dose on airway segmentation. Full-inspiration, full-expiration and half-inflation have been evaluated. In order to evaluate the half-inflation case, a slice thickness of 0.625 mm, high radiation dose and a standard kernel have been chosen. Figure 9 reports the average results obtained across slice thickness and radiation dose for inspiration and expiration phases. Table 8 shows results obtained varying the convolution kernel, whereas in Table 9 parameters and results for the half-inflation dataset are presented.

Although airway segmentation for images acquired during an inspiration phase was better than the segmentation obtained on expiration datasets, in both cases slice thickness is the parameter most affecting the segmentation. In fact, as shown in Figure 9a–c, airway volume, branch count and branch length all increase when thickness is reduced. This result is expected, as less fine details are preserved with thicker slices. At the same time, during inspiration, leakage is more likely when thickness is reduced (see Figure 9d). This is probably due to the higher number of branches segmented, which makes automatic recognition of leakage more complicated. However, it is worth pointing out that, in general, the presence of leakage was insignificant during inspiration, as confirmed by the fact that for a slice thickness of 0.625 mm, an average score around 2 was given to the segmented image. As for the expiration phase, leakage appeared more often than in the inspiration dataset. This is due to the minor presence of air inside the lung, which makes the airways less recognizable from the lung tissues on a CT image. Furthermore, in Figure 9d leakage is increasing for a 2.5 mm slice thickness, with respect to a 5 mm image. However, when thickness is further decreased, the perception of the degree of leakage decreased as well. In this case, the result is probably due to the combination of the thinner slices used (i.e., an image with a better quality) with a smaller number of branches segmented with respect to the inspiration phase. In fact, better quality images enable the segmentation of more branches. At the same time, the segmentation is stopped quite early in this case, thus reducing the probability of leakage.

On the other hand, tube current and radiation dose does not particularly affect airway segmentation, as shown in Figure 9e–h. In this case, for both inspiration and expiration, all the considered metrics do not present a significant variation across the different doses. In particular, the segmented volume does not seem to vary when changing the dose. Branch count and length are slightly increased with increased dose, while leakage occurence is quite stable. Finally, variation of the convolution kernel used for the reconstruction only slightly affects the segmentation for the inspiration phase. As shown in Table 8, a lung kernel allows segmentation of more peripheral branches, at the cost of leakage. However, in general the software can be considered quite stable across kernel variation during an inspiration phase. On the expiration phase, a lung kernel gives better results in terms of volume, branch count and branch length, whereas the kernel does not seem to greatly affect the leakage probability. Good results have also been obtained for half inflation images, as shown in Table 9. In this case, airway segmentation was similar to that obtained for a full-inspiration phase. In general, the results obtained for the breathing pig lung model are quite encouraging and show that the algorithm here proposed can be considered reliable and stable across the different CT acquisition parameters. As expected, slice thickness is the parameter that has the greatest effect on the airway segmentation. However, this is due to the fact that less details are recognizable on the image when thickness is higher. In terms of leakage score, the images were considered mostly highly acceptable, indicating that the algorithm is able to segment as many branches as possible among the ones recognizable on the CT scans, avoiding the leakage appearance.

**Table 9:** Parameters and results for pig lung breathing model during half-inflation

Parameters			Results			
Slice thickness (mm)	Dose	Kernel	Volume (mm^3^)	Branch count	Branch length (mm)	Leakage score
0.625	H	STD	48,171.31	87	2,186.95	2

### Correlation between segmentation metrics and CT acquisition parameters

To improve quantitative analysis of results, we correlated the considered metrics with the different acquisition parameters. This analysis was possible only for the breathing pig lung model, as the clinical case data were too diverse for meaningful comparison. Radiation dose and slice thickness were included. The convolution kernel variation was not included in correlation outputs, as only two values are considered, i.e., lung and standard, and correlation is always maximum as it is easy to understand from Table 8. In fact, all the segmentation measures improved when a lung kernel was used, although the improvement was not significant. Figure 10a shows the correlation for the inspiration case, whereas in Figure 10b correlation during expiration is represented. As seen in Figure 10a, all the segmentation metrics strongly correlated with slice thickness variation for inspiration, confirming the great effect of thickness variation on airway segmentation. In particular, when slice thickness decreased, airway volume, branch count, branch length and leakage increased, giving a correlation value approximately around −1. This result confirms those discussed above. Moreover, as already stated in the previous section, the segmented volume poorly correlated with radiation dose during inspiration, while branch count and length improved in the same way as the dose. Leakage occurence is only slightly correlated to dose variation.

For the expiration case, again slice thickness strongly correlated with volume, branch count, and branch length. However, there is no significant correlation with leakage presence. In terms of dose variation, the metrics correlated as was seen in the inspiration case, with branch count and length following the change of dose more closely than the segmented volume. Moreover, as well as for the slice thickness, leakage was not greatly dependent on the chosen radiation dose. Therefore, these results further confirm that the airway segmentation method proposed here can be considered quite stable across radiation dose and convolution kernel variations. Again, the segmentation greatly depends on the chosen slice thickness, as this substantially affects the quality of the image and the number of branches visible on the CT scan.

As a future evaluation, scanning of a real pig lung has been planned. This would not allow great variation of scanning parameters to test reliability of the algorithm, yet it will give the opportunity to evaluate it on more real images than the one currently available.

## Conclusion

A new semi-automated algorithm for airway segmentation in CT images has been developed. The algorithm is freely available and can be downloaded and used within the 3D Slicer environment. The method is based on a region growing approach, starting from a seed point manually placed within the trachea. The volume is then automatically cropped, and seed points for starting the segmentation are automatically identified within the right and left main bronchi. This way, trachea, right and left bronchial tree can be segmented independently. A method involving the number of the segmented trachea voxels is used in order to stop segmentation before leakage appearance. In the present paper, the performance of the method was qualitatively and quantitatively evaluated on ten human cases belonging to different subjects or scanned with different parameters and forty-eight scans taken from a breathing pig lung model. While direct comparison with other methods is not immediately possible, the aim of this work is for comparative of parameter selection and optimization rather than absolute analysis of segmentation performance. As expected, results show that slice thickness is the parameter which most affects segmentation, whereas variation of radiation dose and convolution kernel do not significantly affect airway segmentation. A CT scan acquired during a full-inspiration breath-hold guarantees a higher contrast between airway and lung tissue, leading to a better segmentation, as proved in both clinical and pig phantom cases. Moreover, results show that the algorithm is able to cease the segmentation before big leakage appears. In general, the results found here are promising, showing reliable methodology stable across varying parameters. Although not optimised to maximise branch detection as other algorithms available in literature, the proposed method allows the segmentation of one lung’s airway regardless of possible leakage appearance in the other. This feature appears novel and avoids early and unnecessary stoppage of the due to leakage. In previous works [12, 17, 38] a predetermined maximum number of voxels or a maximum volume were used as stopping criteria for the segmentation. In the presented work, the maximum number of allowed voxels is determined case by case based on the number of voxels of the trachea segmentation. This gives more flexibility and adaptability to the algorithm. However, in some cases the segmentation does not allow to segment deeper branches, as once the region growing encounters the stopping criteria the segmentation is not further increased. Therefore, the method might benefit of the integration of the region growing with other segmentation techniques, as in [14, 18, 24]. Future improvements will also seek to use different thresholds for different volume of interest within the same lung. Also, performance of the algorithm will be considered on CT images belonging to patients affected by different pulmonary diseases. However, being the first open-source airway-segmentation algorithm available, the proposed approach enables other teams to have a frame of reference to compare segmentation results using personal datasets. Alternatively, the algorithm could be considered as a good starting point for new airway segmentation algorithms that seek to segment narrow peripheral bronchial branches. As a further improvement, we are also expecting to optimize and test the algorithm on more human cases, as well as to scan the pig lung model with different scanner platforms. The final goal is to obtain a method that is stable across all available scanners and allow a stable and reliable segmentation regardless of the parameters chosen for the scanning.
